# Association Between Dexamethasone Treatment After Hospital Discharge for Patients With COVID-19 Infection and Rates of Hospital Readmission and Mortality

**DOI:** 10.1001/jamanetworkopen.2022.1455

**Published:** 2022-03-08

**Authors:** Cheng-Wei Huang, Albert S. Yu, Hubert Song, Joon S. Park, Stefanie S. Wu, Vang Kou Khang, Christopher C. Subject, Ernest Shen

**Affiliations:** 1 Department of Hospital Medicine, Kaiser Permanente Los Angeles Medical Center, Los Angeles, California; 2 Department of Clinical Science, Kaiser Permanente Bernard J. Tyson School of Medicine, Pasadena, California; 3 Department of Internal Medicine, Kaiser Permanente Los Angeles Medical Center, Los Angeles, California; 4 Department of Research and Evaluation, Kaiser Permanente Southern California, Pasadena

## Abstract

**Importance:**

Current guidelines recommend use of dexamethasone, 6 mg/d, up to 10 days or until discharge for patients hospitalized with COVID-19. Whether patients who received less than 10 days of corticosteroids during hospitalization for COVID-19 benefit from continuing treatment at discharge has not been determined.

**Objective:**

To assess whether continuing dexamethasone treatment at discharge is associated with reduced all-cause readmissions or mortality postdischarge.

**Design, Setting, and Participants:**

A retrospective cohort study was conducted at 15 medical centers within Kaiser Permanente Southern California. The population included adults who received less than 10 days of dexamethasone, 6 mg/d, until discharge during hospitalization for COVID-19 and were discharged alive between May 1 and September 30, 2020.

**Exposures:**

Continued dexamethasone treatment at discharge.

**Main Outcomes and Measures:**

All-cause readmissions or mortality within 14 days from discharge.

**Results:**

A total of 1164 patients with a median age of 55 (IQR, 44-66) years were identified. Most patients were of Hispanic ethnicity (822 [70.6%]) and male (674 [57.9%]) and required oxygen support during hospitalization (1048 [90.0%]). Of the 1164 patients, 692 (59.5%) continued dexamethasone, 6 mg/d, at discharge. A balanced cohort was created using propensity score and inverse probability of treatment weighting. The adjusted odds ratio (OR) for readmissions or mortality within 14 days was 0.87 (95% CI, 0.58-1.30) for patients who continued dexamethasone therapy at discharge compared with those who did not. Similar results were produced by a sensitivity analysis that restricted the treatment group to those who received exactly 10 days of dexamethasone (OR, 0.89; 95% CI, 0.55-1.43) and by subgroup analyses stratified by the duration of dexamethasone treatment as an inpatient (1-3 days: OR, 0.71; 95% CI, 0.43-1.16; 4-9 days: OR, 1.01; 95% CI, 0.48-2.12), oxygen requirement at discharge (room air: OR, 0.91; 95% CI, 0.53-1.59; supplemental oxygen use: OR, 0.76; 95% CI, 0.42-1.37), and disease duration at discharge (≤10 days: OR, 0.81; 95% CI, 0.49-1.33; >10 days: OR, 0.94; 95% CI, 0.48-1.86).

**Conclusions and Relevance:**

In this cohort study of patients with COVID-19, continuing treatment with dexamethasone, 6 mg/d, at discharge was not associated with a reduction in 14-day all-cause readmission or mortality. This finding suggests that dexamethasone should not be routinely prescribed beyond discharge for individuals with COVID-19.

## Key Points

**Question:**

Is continuing use of dexamethasone, 6 mg/d, at discharge for patients with COVID-19 who received less than 10 days of dexamethasone treatment during hospitalization associated with readmission or mortality after discharge?

**Findings:**

In a cohort of 1164 patients with COVID-19 who received less than 10 days of dexamethasone, 6 mg/d, during hospitalization, the rate of readmission or mortality within 14 days of discharge was 9.1% among patients who continued dexamethasone treatment compared with 11.4% among patients who did not. The difference was not statistically significant.

**Meaning:**

The findings of this study suggest that prescribing dexamethasone at discharge for patients hospitalized with COVID-19 who received less than 10 days of dexamethasone is not associated with a reduction in readmission or mortality.

## Teaser

This cohort study examines the continued use of dexamethasone therapy following discharge in patients with COVID-19.

## Introduction

Corticosteroids were the first medication class to demonstrate mortality benefit among patients with COVID-19 and are now a cornerstone of COVID-19 treatment.^1,2,3^ In the Randomized Evaluation of COVID-19 Therapy (RECOVERY) trial, the use of dexamethasone, 6 mg/d, up to 10 days or until discharge resulted in lower mortality.^1^ However, with no standardized discharge practices, corticosteroid prescription practices at discharge have varied.

Literature on readmissions following COVID-19 hospitalization have noted that early readmissions after discharge are primarily due to respiratory causes that likely reflect disease progression.^4,5,6,7,8^ Theoretically then, the anti-inflammatory effects of corticosteroids at discharge may reduce such outcomes; a recent study noted that patients who received shorter courses of corticosteroids during hospitalization may be at risk of readmissions and suggested continuation of corticosteroid therapy at discharge.^9^ However, the outcomes associated with continuing corticosteroid treatment at discharge and actual clinical outcomes remain uncertain.

A better understanding of these outcomes, especially in terms of readmissions, would help guide treatment approaches and potentially have a substantial effect on use of resources. In this study, we sought to assess whether continuing corticosteroid treatment at discharge among patients who received less than 10 days of dexamethasone during hospitalization is associated with a reduction in readmissions or mortality following hospitalization due to COVID-19 in a large integrated health system in the US.

## Methods

### Study Design and Setting

This was a retrospective cohort study from 15 medical centers within Kaiser Permanente Southern California (KPSC), which is an integrated health system providing comprehensive coverage and care to more than 4.7 million members throughout Southern California.^10^ The patient population of KPSC is racially and ethnically and socioeconomically diverse, reflecting the general population of Southern California.^11^ The study was approved by the KPSC Institutional Review Board and informed consent was waived owing to the retrospective design. This study followed the Strengthening the Reporting of Observational Studies in Epidemiology (STROBE) reporting guideline for cohort studies.

### Study Population

The study cohort was extracted from an existing cohort of adults (age ≥18 years) discharged alive following hospitalization for COVID-19 between May 1 and September 30, 2020. A COVID-19 hospitalization was defined as an observation stay or inpatient admission with COVID-19 *International Statistical Classification of Diseases, 10th Revision* code U07.1 and a positive SARS-CoV-2 nucleic acid amplification test within 14 days before or up to 48 hours after admission. The first hospital discharge within the study period was defined as the index hospitalization. Exclusion criteria in this existing cohort included COVID-19 hospitalization before the study period in 2020, discharge to hospice, discharge against medical advice, transfer to another acute care hospital, nonmember of KPSC, pregnancy, or hospitalization on a nonmedicine service.

Among these patients, those who received corticosteroids for COVID-19 during the index hospitalization were first identified and those who received only dexamethasone, 6 mg/d, were included in the study. Patients were excluded if they received any corticosteroid other than dexamethasone or any dosage other than 6 mg/d during hospitalization or at discharge, did not receive consecutive doses of dexamethasone up until either the day before or day of discharge, or received 10 days or more of dexamethasone, 6 mg, during hospitalization. Patients were also excluded if any data were missing.

### Data Collection

Kaiser Permanente Southern California has a comprehensive, integrated electronic health records and claims data system that allows for complete data capture both within and outside the system. Race and ethnicity information was available in the electronic health records based on self-report by patients. All variables, including sociodemographic characteristic, comorbidity, clinical, medications, laboratory, and hospitalization and readmission data were collected as part of routine clinical encounters and extracted electronically aside from a subset of patients who had symptom duration obtained via manual medical records review by one of the clinician investigators (C.-W.H., J.S.P., and V.K.K.).

### Exposure Ascertainment

The exposure variable was continuation of dexamethasone at discharge indicated by a filled prescription for dexamethasone, 6 mg/d, at discharge. Duration of the prescription was calculated based on the number and dosage level of the medication.

### Outcome Ascertainment

The outcome was all-cause readmission or mortality within 14 days from discharge. Readmission was defined as an observation stay or inpatient admission following discharge. We selected a 14-day period for our outcome to better reflect readmissions most likely related to COVID-19 and the index hospitalization given reports of early readmissions following hospitalization for COVID-19.^4,6,7^

### Statistical Analysis

Patient characteristics before discharge are first presented descriptively. Continuous variables are presented as median (IQR) and categorical variables as frequencies and percentages.

Propensity score (PS) was used to assess the probability of receiving dexamethasone at discharge. A total of 12 variables detailed in Table 1, including age, sex, race and ethnicity, number of Elixhauser comorbidities, duration of inpatient dexamethasone treatment, other COVID-19 treatments administered, and oxygen status, were used to create the PS using logistic regression. Variables included in PS were selected a priori based on our experience and literature review. Elixhauser score was used as a summative capture of a patient’s comorbidities with higher scores reflecting a greater number of comorbidities.^12^ Duration of inpatient dexamethasone treatment was selected as a variable because shorter courses of corticosteroid therapy may be a risk factor for readmissions and affect duration of treatment prescribed at discharge.^9^ Remdesivir was included because it was the first evidenced-based therapy for COVID-19.^13^ Other therapeutics were included as indirect surrogates for acuity and also to account for any potential confounding. We combined anakinra and tocilizumab into a single variable (ie, biologics) given the limited use of either drug during the study period. Various oxygen-related parameters were included because corticosteroid effects differed by oxygen requirements.^1^ An internal analysis with and without the highest oxygen requirement during hospitalization did not change the study results; therefore, that variable was omitted from the PS to enhance efficiency. In addition, we did not include medical center as a variable within the propensity scoring model or as an adjustment variable in the final logistic regression model to avoid overfitting, and an internal analysis of the intraclass correlation suggested no clustering effects. We used inverse probability of treatment weighting to balance differences in the distributions of these variables between the treatment and control groups. Standardized difference was used to assess the balance between the 2 groups with a difference less than 0.1 considered to be of adequate balance.^14^

**Table 1.** Patient Characteristics Before and at Discharge by Continued Dexamethasone Treatment Before Inverse Probability of Treatment Weighting

Variable	No. (%)		
	All patients (n = 1164)	Dexamethasone (n = 692)	No dexamethasone (n = 472)
Age, median (IQR), y^a^	55 (44-66)	54 (43-65)	57 (47-67.5)
Sex^a^			
Male	674 (57.9)	417 (60.3)	257 (54.4)
Female	490 (42.1)	275 (39.7)	215 (45.6)
Race and ethnicity^a^			
Asian	110 (9.5)	73 (10.5)	37 (7.8)
Black	86 (7.4)	49 (7.1)	37 (7.8)
Hispanic	822 (70.6)	491 (71)	331 (70.1)
White	146 (12.5)	79 (11.4)	67 (14.2)
BMI, median (IQR)^a^	31.3 (27.5-36.3)	31.4 (27.5-36.2)	31.2 (27.5-36.6)
No. of Elixhauser comorbidities, median (IQR)^a^^,^^b^	3 (1-4)	3 (1-4)	3 (2-5)
Elixhauser comorbidities			
≤3	740 (63.6)	690 (65.9)	50 (42.7)
>3	424 (36.4)	357 (34.1)	67 (57.3)
Inpatient dexamethasone treatment, median (IQR), d^a^	4 (3-6)	4 (3-6)	4 (2-6)
Other COVID-10 treatment			
Remdesivir^a^	631 (54.2)	352 (50.9)	279 (59.1)
Convalescent plasma^a^	153 (13.1)	81 (11.7)	72 (15.3)
Biologics^a^^,^^c^	59 (5.1)	51 (7.4)	8 (1.7)
Therapeutic anticoagulation^a^	187 (16.1)	112 (16.2)	75 (15.9)
Highest O_2_ requirement			
Room air	116 (10)	62 (9)	54 (11.4)
Supplemental oxygen	894 (76.8)	544 (78.6)	350 (74.2)
High-flow/NIPPV	109 (9.4)	59 (8.5)	50 (10.6)
Mechanical ventilation	45 (3.9)	27 (3.9)	18 (3.8)
At discharge			
Supplemental O_2_ required^a^	558 (47.9)	338 (48.8)	220 (46.6)
Hypoxia (O_2_ saturation <94%)^a^	253 (21.7)	161 (23.3)	92 (19.5)
Symptom duration ≤10 d^a^^,^^d^	396 (34)	227 (32.8)	169 (35.8)

Abbreviations: BMI, body mass index (calculated as weight in kilograms divided by height in meters squared); NIPPV, noninvasive positive pressure ventilation.

^a^ Included in propensity score.

^b^ Categorical values were used in creating the propensity score.

^c^ Included both anakinra and tocilizumab.

^d^ Symptom onset date was defined as day 1. Obtained by manual review of medical records (n = 117); admission date was used as the symptom onset date for patients who were not identifiable as having symptomatic COVID-19 before hospitalization (n = 39).

A logistic regression was then performed to estimate the odds ratio (OR) and 95% CI of 14-day all-cause readmission or mortality with continuation of dexamethasone treatment at discharge as the exposure variable. Additional adjustment for symptom duration—a dichotomous variable categorized as 10 days or less and more than 10 days at discharge—was then performed. We did not include symptom duration in the PS because we did not believe that it was routinely factored into decision-making for prescribing corticosteroids at discharge but adjusted for it in the regression because it may have the potential to affect the study outcome given our clinical experience.

To assess the robustness of our findings, we performed a sensitivity analysis restricting the treatment group to patients who received exactly 10 days of dexamethasone therapy based on the sum of their days of inpatient dexamethasone treatment and days of dexamethasone prescribed at discharge.

We also performed 3 exploratory subgroup analyses stratified by days of inpatient dexamethasone treatment, oxygen requirement at discharge, and symptom duration to assess for potential effect modification. The optimal cutoff points for the subgroup analysis examining the days of inpatient dexamethasone treatment was derived using both the Youden J Index and the minimization of the Euclidean distance for the sensitivity and specificity from the point (0, 1).

For each sensitivity and subgroup analysis, a PS was recalculated for each comparison followed by inverse probability of treatment weighting to obtain a balanced treatment and control group in our logistic regression models that otherwise mimic the main analysis. All statistical analyses were conducted in SAS, version 9.4 (SAS Institute Inc). A 2-sided *P* value <.05 was considered statistically significant.

## Results

**Figure 1.**
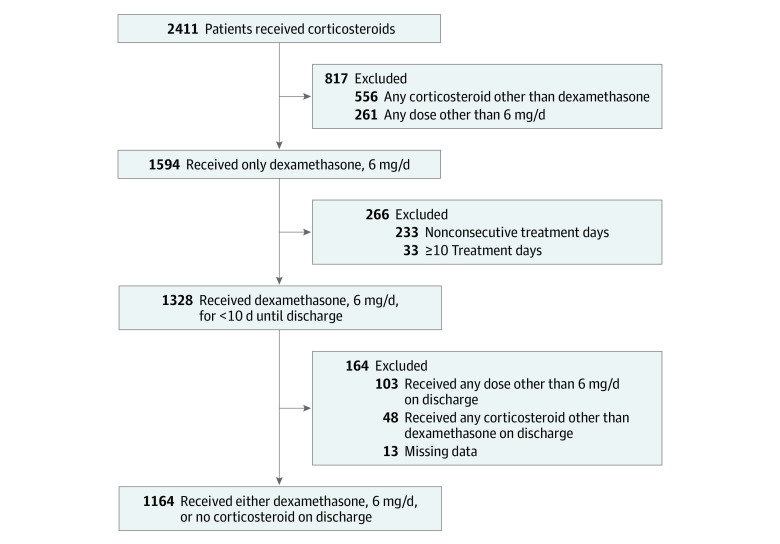
Population Flowchart Data were missing on race and ethnicity (n = 7), body mass index (n = 2), and oxygen requirement at discharge (n = 4).

A cohort of 1164 patients with COVID-19 who received less than 10 days of dexamethasone, 6 mg/d, therapy until discharge was identified; all patients were discharged after June 16, 2020, when the results of the RECOVERY trial were first released (Figure 1).^15^ The median age was 55 (IQR, 44-66) years; 674 patients were men (57.9%) and 490 (42.1%) were women. Race and ethnicity groups represented were Asian (110 [9.5%]), Black (86 [7.4%]), Hispanic (822 [70.6%]), and White (146 [12.5%]). Most patients (1048 [90.0%]) required some form of oxygen support during hospitalization. The median duration of inpatient dexamethasone treatment was 4 (IQR, 3-6) days across the entire cohort.

Of the 1164 patients, 692 (59.5%) continued dexamethasone therapy at discharge. The median duration of dexamethasone treatment at discharge was 5 (IQR, 4-7) days. The median total duration of dexamethasone therapy (including both days during hospitalization and at discharge) in this treatment group was 10 (IQR, 10-11) days. Among patients who continued dexamethasone therapy, 63 (9.1%) patients were readmitted or died within 14 days at a median time of 3 (IQR, 1-5) days from discharge. Among patients who did not continue dexamethasone therapy, 54 (11.4%) individuals were readmitted or died within 14 days at a median time of 3 (IQR, 2-5) days from discharge. Outcomes in the 62 patients (98.3%) who continued dexamethasone treatment can be attributed to readmissions compared with 52 (96.3%) in those who discontinued dexamethasone (eTable 1 in the Supplement).

Before inverse probability of treatment weighting, the group that continued dexamethasone therapy vs those who did not comprised fewer women (275 [39.7%] vs 215 [45.6%]) and fewer patients with more than 3 Elixhauser comorbidities (357 [34.1%] vs 67 [57.3%]). Patients who continued dexamethasone treatment also were less likely to have received remdesivir (352 [50.9%] vs 279 [59.1%]) but more likely to have received biologics (51 [7.4%] vs 8 [1.7%]). After inverse probability of treatment weighting, the standardized differences between the 2 groups for all 12 variables included in the PS were within 0.1, suggesting that the 2 groups were well balanced (eTable 2 in the Supplement).

After inverse probability of treatment weighting, the adjusted OR of 14-day readmission or mortality was 0.87 (95% CI, 0.58-1.30) for patients who continued dexamethasone therapy compared with those who discontinued dexamethasone (Table 2). The results were similar in a sensitivity analysis that restricted the treatment group to those who received exactly 10 days of dexamethasone, with all standardized differences between the 2 groups within 0.1 (OR, 0.89; 95% CI, 0.55-1.43) (Table 2).

**Table 2.** Adjusted Odds Ratio of 14-Day All-Cause Readmission or Mortality Among Patients Continuing Dexamethasone at Discharge After Inverse Probability of Treatment Weighting

Characteristic	No. of patients	Adjusted odds ratio (95% CI)^a^
**Main analysis**		
Continued dexamethasone at discharge	692	0.87 (0.58-1.30)
Discontinued dexamethasone at discharge	472	1 [Reference]
**Sensitivity analysis**		
Received exactly 10 d of dexamethasone	350	0.89 (0.55-1.43)
Discontinued dexamethasone at discharge	472	1 [Reference]

^a^ Odds ratios were calculated with additional adjustment of symptom duration at discharge (<10 days) after inverse probability treatment weighting of propensity score, including age, sex, race and ethnicity, body mass index, number of Elixhauser comorbidities (category), inpatient dexamethasone treatment (days), remdesivir therapy, convalescent plasma, biologics therapy (including anakinra and tocilizumab), therapeutic anticoagulation, supplemental oxygen at discharge, and hypoxia (O_2_ saturation <94%) at discharge.

**Figure 2.**
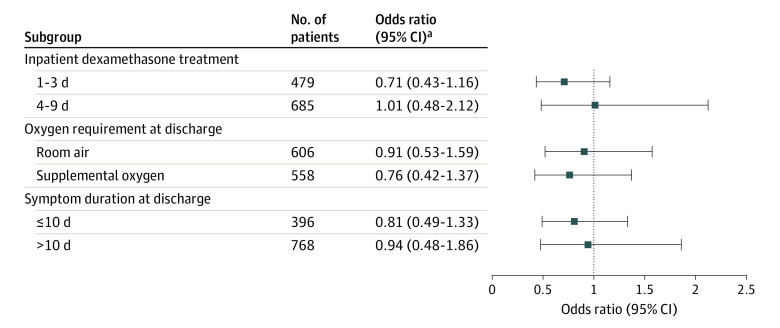
Adjusted Odds Ratios of 14-Day All-Cause Readmission or Mortality by Subgroup After Inverse Probability of Treatment Weighting An odds ratio less than 1 favors continuing dexamethasone at discharge. ^a^The variable for subgroup stratification was removed from the adjustment or propensity score. Otherwise, odds ratios were calculated with additional adjustment of symptom duration at discharge (<10 days) after inverse probability treatment weighting of propensity score including age, sex, race and ethnicity, body mass index, number of Elixhauser comorbidities (category), duration of inpatient dexamethasone treatment (days), remdesivir treatment, convalescent plasma, biologics therapy (including anakinra and tocilizumab), therapeutic anticoagulation, supplemental oxygen at discharge, and hypoxia (O_2_ saturation <94%) at discharge.

In the subgroup analysis stratified by days of inpatient dexamethasone treatment, a cutoff of 3 days was identified using the Youden J index and also minimization of the Euclidean distance for the sensitivity and specificity from the point (0, 1), which was also consistent with a subgroup cutoff within a previous report.^9^ The results were largely consistent across each subgroup analysis with all standardized differences for each subgroup analysis within 0.1, with duration of dexamethasone treatment as an inpatient (1-3 days: OR, 0.71; 95% CI, 0.43-1.16; 4-9 days: OR, 1.01; 95% CI, 0.48-2.12), oxygen requirement at discharge (room air: OR, 0.91; 95% CI, 0.53-1.59; supplemental oxygen: OR, 0.76; 95% CI, 0.42-1.37), and disease duration at discharge (≤10 days: OR, 0.81; 95% CI, 0.49-1.33; >10 days: OR, 0.94; 95% CI, 0.48-1.86) (Figure 2).

## Discussion

In a diverse cohort of patients with COVID-19 who received less than 10 days of treatment with dexamethasone, 6 mg/d, during COVID-19 hospitalization, we found that continuing dexamethasone therapy at discharge was not associated with a reduction in 14-day readmission or mortality postdischarge. These findings were largely consistent across varying durations of inpatient dexamethasone treatment, symptom durations at discharge, and oxygen requirements at discharge. Studies exploring the utility of continuing dexamethasone therapy at discharge have been sparse and our study adds novel insights and evidence regarding the appropriate treatment of COVID-19. The findings also raise questions regarding the prevailing practice of continuing corticosteroid therapy at discharge despite guideline recommendations.^2,3^

Our findings have important implications for clinical practice. Our study, which spanned a period of the 2020 summer/fall surge in COVID-19 cases in Southern California, suggests that once a patient’s condition is stable for discharge, continuing dexamethasone therapy does little to alter the natural course of the disease. Adverse effects of corticosteroids, including opportunistic infections and hyperglycemia, have been well described.^16^ In other diseases, such as chronic obstructive pulmonary disease, studies over time have reported that shorter courses of corticosteroids were noninferior to longer courses.^17^ Avoiding nonbeneficial use of corticosteroids and limiting to what is necessary would be consistent with the mantra of do no harm.

Although our study was done early in the pandemic, our findings remain of relevance in today’s practice, especially because corticosteroids have become a cornerstone therapy for COVID-19. Studies have suggested a decrease in poor outcomes, such as mortality, in patients with COVID-19 as the pandemic progressed.^18,19,20^ We believe that a negative study finding early in the pandemic would apply even more so today because poor outcomes are less likely with better understanding of the disease, increased levels of vaccination, and variants that result in diseases with lower acuity.^21,22^

There are at least 2 plausible explanations for our findings. The longer half-life of dexamethasone and self-tapering effect may have reduced any significant differences observed with extending the corticosteroid course. Whether the same findings would hold true for other corticosteroids with differing pharmacokinetics is unknown. Alternatively, it may be that any benefit derived from a reduction in COVID-19 disease severity is offset by potential complications of continuing the corticosteroid course; however, we did not explore specific reasons for readmissions in our study. Better understanding of potential complications in this population may be an area of future investigation.

Our findings do not necessarily conflict with existing literature. Chaudhry et al^9^ had noted that patients who received dexamethasone for only 1 to 3 days during hospitalization were most at risk for readmissions. We observed a relatively lower point estimate with continuing dexamethasone treatment among those who received dexamethasone for 1 to 3 days vs 4 to 9 days while patients were hospitalized and those who had symptom durations of 10 days or less vs more than 10 days at discharge. These observations may suggest that patients could have derived some benefit early in the disease course from continuing dexamethasone at discharge. Subgroup analysis should be interpreted with caution owing to the potential for false-negative and false-positive findings.^23^

### Strengths and Limitations

Our study has many strengths, including a diverse cohort and comparative analysis with clinical outcomes. We were able to confirm our findings across multiple analyses and explore specific clinical conditions, including oxygen requirements at discharge, which has been used widely as a surrogate for corticosteroid use.^1^ Our exploratory subgroup analysis showed a relatively lower point estimate for patients who had received 1 to 3 days of inpatient dexamethasone treatment, and further studies regarding patients earlier in the disease course and/or those who have received a shorter duration of corticosteroid therapy before discharge are needed.

The study has limitations. Despite a well-balanced cohort with inverse probability of treatment weighting, our study remained susceptible to possible indication biases as well as unadjusted confounders, such as our lack of data on corticosteroid use before hospitalization. We also did not have direct measures for disease severity during the index hospitalization other than oxygen requirement. However, we had other indirect measures, such as use of biologics and therapeutic anticoagulation, which were more likely to be given in patients with higher acuity levels, as well as duration of inpatient dexamethasone treatment, which should in part reflect length of stay. Moreover, our exposure was based on prescription data, and we were unable to confirm adherence. Oxygen requirements and saturations were based on a single reading and may not always reflect a patient’s true clinical status. Our findings may not be generalizable to use of other corticosteroids or dosing regimens, given the difference in pharmacokinetics. We did not explicitly account for loss of follow-up because only KPSC members were included in the study; however, any lost data should be minimal given the short follow-up period. There is always the possibility that we had a false-negative study, and our findings should be further investigated. In addition, we lacked details on the cause of readmissions and could not assess whether readmissions were due to a lack of reduction in COVID-19 disease progression or increased complications secondary to corticosteroid use. However, even if there was some reduction in disease progression, our overall negative finding would argue that the benefits of continuing dexamethasone treatment at discharge do not outweigh the risks.

## Conclusions

In this cohort study of patients with COVID-19 infection discharged from hospitals, continuing dexamethasone, 6 mg/d, at discharge for patients who received less than 10 days of dexamethasone, 6 mg/d, during hospitalization was not associated with a reduction in 14-day readmissions or mortality. Our findings suggest that dexamethasone, short of other indications, should not be routinely prescribed beyond discharge for treatment in patients with COVID-19.

## Notes

Supplement. **eTable 1.** Number of Events by Continued Dexamethasone Treatment on Discharge **eTable 2.** Standardized Differences Before and After Inverse Probability of Treatment Weighting for All Variables
