# Numeric Errors in Table 1

**DOI:** 10.1001/jamanetworkopen.2022.16873

**Published:** 2022-05-20

In the Original Investigation titled “Association Between Dexamethasone Treatment After Hospital Discharge for Patients With COVID-19 Infection and Rates of Hospital Readmission and Mortality,”^1^ published March 8, 2022, there were errors in the numbers and percentage values in Table 1 for the rows “Supplemental oxygen,” “High-flow/NIPPV,” and “Mechanical ventilation.” This article has been corrected.^1^
