# Supplementary material for: Association Between Dexamethasone Treatment After Hospital Discharge for Patients With COVID-19 Infection and Rates of Hospital Readmission and Mortality
Source: JAMA Netw Open. 2022 Mar 8;5(3):e221455. doi: 10.1001/jamanetworkopen.2022.1455 (PMC8905383; doi:10.1001/jamanetworkopen.2022.1455)
Supplement: Supplement. — eTable 1. Number of Events by Continued Dexamethasone Treatment on Discharge eTable 2. Standardized Differences Before and After Inverse Probability of Treatment Weighting for All Variables [file jamanetwopen-e221455-s001.pdf]

Supplementary Online Content

Huang CW, Yu AS, Song H, et al. Association between dexamethasone treatment after hospital discharge for patients with COVID-19 infection and rates of hospital readmission and mortality. *JAMA Netw Open*. 2022;5(3):e221455. doi:10.1001/jamanetworkopen.2022.1455

eTable 1. Number of Events by Continued Dexamethasone Treatment on Discharge

eTable 2. Standardized Differences Before and After Inverse Probability of Treatment Weighting for All Variables

This supplementary material has been provided by the authors to give readers additional information about their work.

© 2022 Huang CW et al. *JAMA Network Open.*

eTable 1. Number of events by continued dexamethasone treatment on discharge[a]

**Event[b]**	**All patients (n=1164)**	**Dexamethasone (n= 692)**	**No dexamethasone (n= 472)**
Readmission or mortality			
No. of events	117 (10.1%)	63 (9.1%)	54 (11.4%)
Days to event	3 (1, 5)	3 (1, 5)	3 (2, 5)
Readmission			
No. of events	114 (9.8%)	62 (9.0%)	52 (11%)
Days to event	3 (1, 5)	3 (1, 5)	2.5 (2, 4.5)
Mortality			
No. of events	10 (0.9%)	6 (0.9%)	4 (0.8%)
Days to event	7 (5, 10)	8 (5, 13)	7 (5, 7.5)

[a]Data presented as absolute number (%) and median (interquartile range).

[b]Readmission and mortality are not mutually exclusive and therefore total number may exceed that of readmission or mortality.

© 2022 Huang CW et al. *JAMA Network Open.*

eTable 2. Standardized differences before and after inverse probability of treatment weighting for all variables

Variable	Before IPTW	After IPTW
1. Age (year)	**-0.1393**	-0.0076
2. Sex	**0.1177**	-0.008
3. Race/Ethnicity	**0.1222**	0.023
4. Body mass index (+1)	0.0232	0.0283
5. Elixhauser Index (category)	**-0.141**	0.0073
6. Dexamethasone treatment inpatient (days)	0.0706	-0.0101
7. Remdesivir	**-0.1663**	0.0058
8. Convalescent plasma	**-0.1041**	0.0135
9. Biologics including anakinra and tocilizumab	**0.2754**	-0.0115
10. Therapeutic anticoagulation	0.008	-0.0175
11. Oxygen required at discharge	0.0447	-0.0146
12. Hypoxia (<94%) at discharge	0.0922	0.0132

© 2022 Huang CW et al. *JAMA Network Open.*
